# Bibliography

**Published:** 1890-07

**Authors:** 


### BIBLIOGRAPHY.

Annual of the Universal Medical Sciences. Charles E. Sajous, M.D., and Seventy Associate Editors. Philadelphia: F. A. Davis, Publisher.

The *Annual*, although delayed some twenty days by the hold La Grippe had upon the editorial staff, appears bright and fully up to its former standard, and gives no evidence of lessened vitality in its editing or publication. On the other hand, several new departments have been added: One on Syphilis, by J. William White, Philadelphia, and another on Surgical Mycoses, by Ernest Laplace, also of Philadelphia. The department of Oral Surgery has been given to R. Matas, New Orleans, who has handled the subject in a masterly manner.

This chapter alone is worth the subscription-price of the work to all dentists who have a liking for that branch of dental practice. On the whole, the work is invaluable as a review of the very latest work done in medical science, pointing out as it does the best line of thought presented during the past year in all the different countries of the world. Reference is made to the authority in every instance, thus enabling the reader to seek the original and read the article in its entirety, if it is desired so to do. Forming as it does a complete *résumé* of scientific progress in medicine, we do not see how any progressive man can do without it, especially if he desires to write for publication. W. X. S.

PHYSIOLOGY OF THE DOMESTIC ANIMALS. A Text-Book for Veterinary and Medical Students and Practitioners. By ROBERT MEADE SMITH, A.M., M.D., Professor of Comparative Physiology in the University of Pennsylvania. Pp. 938. Price, cloth, $5.00; sheep, $6.00 net. Philadelphia: F. A. Davis, publisher.

The author of this volume has spared neither time nor pains to make it of value to those interested in the important subject of physiology. His matter is arranged in a logical manner; Part I. is devoted to General Physiology; Part II. to Special Physiology; and Part III. to the Reproductive Functions. Over four hundred good illustrations adorn the work and add light to the contents.

In these days of multiplication of books it is seldom that one can be written which may be truly said to fill a want. But up to this time there has not been in English a work of this special character, and as the great importance of comparative physiology in the study of that pertaining to man can hardly be overestimated, we heartily welcome the advent of this book. W. X. S.

